# Moral Enhancement

**DOI:** 10.1111/j.1468-5930.2008.00412.x

**Published:** 2008-08

**Authors:** Thomas Douglas

**Affiliations:** Faculty of Philosophy and Christ Church, University of OxfordSt Aldates, Oxford OX1 1DP, UK

## Abstract

Opponents of biomedical enhancement often claim that, even if such enhancement would benefit the enhanced, it would harm others. But this objection looks unpersuasive when the enhancement in question is a moral enhancement — an enhancement that will expectably leave the enhanced person with morally better motives than she had previously. In this article I (1) describe one type of psychological alteration that would plausibly qualify as a moral enhancement, (2) argue that we will, in the medium-term future, probably be able to induce such alterations via biomedical intervention, and (3) defend future engagement in such moral enhancements against possible objections. My aim is to present this kind of moral enhancement as a counter-example to the view that biomedical enhancement is always morally impermissible.

Biomedical technologies are routinely employed in attempts to maintain or restore health. But many can also be used to alter the characteristics of already healthy persons. Without thereby attributing any value to these latter alterations, I will refer to them as *biomedical enhancements*.

Biomedical enhancement is perhaps most apparent in sport, where drugs have long been used to improve performance,^1^ but it is also widespread in other spheres. Some musicians take beta-blockers to calm their nerves before performances,^2^ a significant proportion of American college students report taking methylphenidate (Ritalin) while studying in order to improve performance in examinations,^3^ and then, of course, there is cosmetic surgery. Research on drugs that may enhance memory,^4^ the retention of complex skills,^5^ and alertness^6^ suggests that the possibilities for biomedical enhancement are likely to grow rapidly in coming years. However, the *morality*of using biomedical technologies to enhance remains a matter of controversy. Some argue that it would be better if people were more intelligent, longer-lived, and physically stronger, and that there is no objection to using biomedical technologies to achieve these goals. But others hold that biomedical enhancement ought to be avoided.

## The Bioconservative Thesis

The opponents of enhancement do not all set out to defend a common and clearly specified thesis. However, several would either assent or be attracted to the following claim (henceforth, the Bioconservative Thesis):

Even if it were technically possible and legally permissible for people to engage in biomedical enhancement, it would not be *morally permissible*for them to do so.^7^

The scope of this thesis needs to be clarified. By ‘people’, I mean here to include all currently existing people, as well as those people that may exist in the medium term future — say, the next one hundred years — but not people who may exist in the more distant future. Similarly, I mean to include under ‘biomedical enhancement’ only those enhancement practices that may plausibly become technically feasible in the medium term future. The opponents of enhancement may justifiably have little to say about enhancements that would take place in the distant future, or would require far-fetched technologies.

In what follows, I argue that the Bioconservative Thesis, thus qualified, is false.

## A Possible Counter-Example to the Bioconservative Thesis

The Bioconservative Thesis may be defended in various ways. But many of the most prevalent arguments for it are based on social considerations: though enhancement might be good for the enhanced individuals, it could well be bad for others.^8^ Thus, regarding intelligence enhancement it could be argued that if one person makes herself more intelligent she will disadvantage the unenhanced by, for example, out-competing them for jobs, or by discriminating against them on the basis of their lower intelligence.^9^

These arguments may be persuasive when directed against the most commonly discussed biomedical enhancements — physical ability enhancements, intelligence and memory enhancements, and natural lifespan enhancements. But there are other types of biomedical enhancement against which they appear much less persuasive. In this paper I will focus on one possibility: that future people might use biomedical technology to *morally* enhance themselves.

There are various ways in which we could understand the suggestion that we morally enhance ourselves. To name a few, we could take it as a suggestion that we make ourselves more virtuous, more praiseworthy, more capable of moral responsibility, or that we make ourselves act or behave more morally. But I will understand it in none of these ways. Rather, I will take it as a suggestion that we cause ourselves to have morally better motives (henceforth often omitting the ‘morally’). I understand motives to be the psychological — mental or neural — states or processes that will, given the absence of opposing motives, cause a person to act.^10^

Since I focus only on motives, I will not claim that the morally enhanced person *is*more moral, has a more moral character, or will necessarily act more morally than her earlier, unenhanced self. I will also try to avoid committing myself to any particular view about what determines the moral goodness of a motive. For example, I will, insofar as possible, remain neutral between the views that the moral goodness of a motive is determined by the sort of acts it motivates, the character traits it partially constitutes, the consequences of its existence, or its intrinsic properties.

With these qualifications in hand, I now set out my formula for moral enhancement:

A person morally enhances herself if she alters herself in a way that may reasonably be expected to result in her having morally better future motives, taken in sum, than she would otherwise have had.

This formula strikes me as a natural way of capturing the idea of moral enhancement given our focus on the moral goodness of motives. However, it has three noteworthy features. First, it compares *sets*of motives, rather than individual motives. More specifically, it compares the full set of future motives that an agent would have following enhancement with the one he would have without it. Second, it focuses on whether an alteration may *reasonably be expected* to result in the agent having morally better motives (or, as I will henceforth say, whether it will *expectably* lead the agent to have better motives), not on whether it actually succeeds in bringing about those motives. Without this second condition it would be difficult to know in advance whether some alteration would constitute a moral enhancement. Third, my formula allows that a moral enhancement may be achieved through non-biomedical means. I will focus specifically on the case of *biomedical*moral enhancement later.

Unlike the most frequently mentioned varieties of enhancement, enhancements satisfying this formula for moral enhancement could not easily be criticised on the ground that their use by some would disadvantage others. On any plausible moral theory, a person's having morally better motives will tend to be to the advantage of others. Indeed, on some views, the fact that having some motive would tend to advantage others is what makes it a morally good motive. Admittedly, acquiring a better set of future motives may sometimes cause a person to inflict disadvantage on other persons, but it will do this only when (a) the better motives fail to have their typical effects (as where an appropriate desire to help others has, due to unforeseen circumstances, harmful effects), (b) the disadvantage serves some moral purpose (as where a concern for justice leads someone to inflict an appropriate punishment on a wrongdoer), or (c) having a morally better overall set of future motives involves having some worse individual motives. One could not object to moral enhancement on the ground that it would systematically impose morally gratuitous disadvantage on others.

Indeed, I will argue that, when performed under certain conditions, there would be no good objection — social or other — to biomedical moral enhancement. I will suggest that it would, contrary to the Bioconservative Thesis, be morally permissible for people to undergo such enhancements. Before proceeding to my argument, however, it is necessary to say something more about how moral enhancement might work.

## The Nature of Moral Enhancement

There is clearly scope for most people to morally enhance themselves. According to every plausible moral theory, people often have bad or suboptimally good motives. And according to many plausible theories, some of the world's most important problems — such as developing world poverty, climate change and war — can be attributed to these moral deficits.

But it is not immediately clear what sorts of psychological changes would count as moral enhancements. There are at least two reasons for the lack of clarity.

First, there is little agreement on which motives are morally good and to what degree. Whereas some would claim that it is best to be motivated by normative beliefs formed as a result of correct reasoning processes,^11^ others would emphasise the importance of moral emotions such as sympathy.^12^ Others still would favour some mixture of the two.^13^ Moreover, this disagreement cannot be resolved by appealing to some view about what sorts of consideration determine the moral goodness of a motive, since here there is even less agreement. For example, some would hold that a motive is morally good to the extent that it tends to produce good consequences, while others would hold that motives are good to the extent that they are partly constitutive of certain virtues.

Second, both what counts as a good motive and what counts as an improvement in one's motives will be different for different people, or people performing different roles. For a judge, a certain sort of legal reasoning might be the best motive, whereas for a parent, love might be more appropriate. Similarly, for a person who feels little sympathy for others, an increase in sympathy might count as a moral improvement. But for someone who is already overwhelmed by feelings of sympathy, any such increase is unlikely to count as an improvement.

Despite these difficulties, I think it would be possible to identify several kinds of psychological change that would, for some people under some circumstances, uncontroversially qualify as moral enhancements. I will focus solely on one possibility here. My thought is that there are some emotions — henceforth, the counter-moral emotions — whose attenuation would sometimes count as a moral enhancement regardless of which plausible moral and psychological theories one accepted. I have in mind those emotions which may interfere with all of the putative good motives (moral emotions, reasoning processes, and combinations thereof) and/or which are themselves uncontroversially *bad*motives. Attenuating such emotions would plausibly leave a person with better future motives, taken in sum.

One example of a counter-moral emotion might be a strong aversion to certain racial groups. Such an aversion would, I think, be an uncontroversial example of a bad motive. It might also *interfere with*what would otherwise be good motives. It might, for example, lead to a kind of subconscious bias in a person who is attempting to weigh up the claims of competing individuals as part of some reasoning process. Alternatively, it might limit the extent to which a person is able to feel sympathy for a member of the racial group in question.

A second example would be the impulse towards violent aggression. This impulse may occasionally count as a good motive. If I am present when one person attacks another on the street, impulsive aggression may be exactly what is required of me. But, on many occasions, impulsive aggression seems to be a morally bad motive to have — for example, when one has just been mildly provoked. Moreover, as with racial aversion, it could also interfere with good motives. It might, for example, cloud a person's mind in such a way that reasoning becomes difficult and the moral emotions are unlikely to be experienced.

I suspect, then, that for many people the mitigation of an aversion to certain racial groups or a reduction in impulsive violent aggression would qualify as a moral enhancement — that is, it would lead those people to expectably have better motives, taken in sum, than they would otherwise have had. However, I do not want, or need, to commit myself to this claim here. Rather, I will stake myself to the following weaker claim: there are some emotions such that a reduction in the degree to which an agent experiences those emotions would, under some circumstances, constitute a moral enhancement.

Two broadly Kantian objections might be made to this claim. First, it might be objected that when a person brings about certain motives in herself, the moral goodness of those motives is wholly determined by the earlier motives for bringing them about. The locus of moral appraisal is shifted from the (later) motives that are brought about to the (earlier) motives for bringing them about. Thus, though it might normally be true that the attenuation of some emotion would improve one's motives, this will not necessarily be the case when the attenuation of the emotion is itself a motivated action. If, say, one is motivated to alter one's emotions by some bad motive, the badness of the earlier motive may infect the subsequent motives.

It is implausible, however, that the goodness of a person's motives at some time is determined wholly by the earlier motives that brought those later motives about. Suppose that a neo-Nazi attends an anti-Semitic rally in order to protest against an influx of Jews into his city. But suppose that he is, unexpectedly, sickened by the behaviour of his co-protestors, and impressed by the conduct of the horrified Jewish onlookers. The upshot is that he is left with a greatly diminished aversion to Jewish people. Intuitively, this person has better motives after the rally than he had before. But it is difficult to see how this improvement in his motives could be explained by reference to the motives which brought it about. Those earlier motives were, after all, racist ones.

A second objection to my account of moral enhancement would maintain that nothing which alters only emotions could truly give an agent better motives. The only thing susceptible of moral appraisal is, it might be argued, the will. Thus, the only motives capable of being good or bad are those that consist in the exercise of the will. And whether one experiences certain emotions or not is simply irrelevant to the question whether one has such motives, for emotions lie outside of the boundaries of the will. Rather, the will is exercised through engagement in reasoning processes that are independent of emotional states: these reasoning processes are the only motives that can be good or bad (they will be good, on the Kantian view, when they are properly directed by the moral law — or, as I will henceforth say, when they are *correct*).^14^

The view that reasoning processes are the only motives susceptible of moral appraisal strikes me as implausible. Intuitively one can sometimes morally improve one's motives by, for example, cultivating feelings of sympathy. But this could not count as an improvement on the Kantian view just sketched, since being moved by sympathy surely does not count as engaging in reasoning. Moreover, even if we accept that reasoning processes are the only motives susceptible of moral appraisal, *attenuating* an emotion might still count as a moral enhancement. Though emotions may lie outside the will, they may interfere with its exercise by corrupting reasoning processes. Thus, attenuating the problematic emotions may allow an agent to engage in correct practical reasoning processes when that would not otherwise have been possible.

There is, it must be admitted, a stronger version of the Kantian position. It could be argued that one exercises one's will only when one engages in reasoning processes that are *insusceptible to emotional interference.*On this view, even though attenuating counter-moral emotions might enable an agent to engage in correct reasoning processes, those processes could not themselves count as good (or bad) motives, precisely because they were susceptible to emotional interference.

I cannot adequately respond to this objection here. However, since I doubt that many people will subscribe to the strong view about the nature of the will that it presupposes, I am not sure that any response is called for. I will simply record that, like the weaker Kantian view, this stronger view implies that cultivating certain emotions cannot in any way morally improve one's motives. Unlike the weaker view, it also implies (in my view counter-intuitively) that neither training oneself to suppress emotions such as racial aversion, nor avoiding circumstances known to provoke them, could affect the goodness of one's motives.

## The Possibility of Biomedical Moral Enhancement

I will tentatively argue that it would sometimes be morally permissible for people to biomedically mitigate their counter-moral emotions. But first I want to briefly consider what might appear to be a prior question. Will this sort of biomedical moral enhancement be possible within the medium-term time span that we are considering?

There are two obvious reasons for doubting that biomedical moral enhancement will, in the medium term, become possible. The first is that there are, on some views about the relationship between mind and brain, some aspects of our moral psychology that cannot in principle be altered through biological intervention.^15^ This is not the place to explore this claim. I hope it suffices merely to note that it is not a mainstream philosophical position. The second ground for doubt is that our moral psychology is presumably highly complex — arguably, so complex that we will not, within the medium term future, gain sufficient understanding of its neuroscientific basis to allow the informed development of appropriate biomedical interventions.

There are surely some aspects of our moral psychology that are exceedingly complex. We probably will not, in the medium term, properly understand the neuroscientific basis of belief in Kant's categorical imperative. But there are other elements of our moral psychology that may be more amenable to understanding, and these would plausibly include at least some of the counter-moral emotions.

Consider the two emotions that I mentioned earlier — aversion to certain racial groups, and impulses towards violent aggression. Work in behavioural genetics and neuroscience has lead to an early but growing understanding of the biological underpinnings of both. There has long been evidence from adoption and twin studies of a genetic contribution to aggression,^16^ and there is now growing evidence implicating a polymorphism in the Monoamine Oxidase A gene,^17^ and, at the neuro-physiological level, derangements in the serotonergic neurotransmitter system.^18^ Race aversion has been less well studied. However, a series of recent functional magnetic resonance imaging studies suggest that the amygdala — part of the brain already implicated in the regulation of emotions — plays an important role.^19^ Given this progress in neuroscience, it does not seem unreasonable to suppose that moral enhancement technologies which operate on relatively simple emotional drives could be developed in the medium term.

## The Scenario

I am now in a position to set out the conditions under which it would, I will argue, be morally permissible for people to morally enhance themselves. These conditions are captured in a scenario consisting of five assumptions.^20^

The first assumption simply specifies that we are dealing with an enhancement that satisfies my formula for moral enhancement:

**Assumption 1.** Through undergoing some biomedical intervention (for example, taking a pill) at time *T*, an agent Smith can bring it about that he will expectably have better post-*T* motives than he would otherwise have had.

In order to focus on the situation where the case for moral enhancement is, I think, strongest, I introduce a second assumption as follows:

**Assumption 2.**If Smith does not undergo the intervention, he will expectably have at least some bad (rather than merely suboptimally good) motives.

A third assumption captures my earlier claim about how, as a matter of psychology, moral enhancement might work:

**Assumption 3.** The biomedical intervention will work by attenuating some emotion(s) of Smith's.

And finally, the fourth and fifth assumptions rule out what I take to be uninteresting objections to moral enhancement: that it might have adverse side effects, and that it might be done coercively or for other unnecessarily bad reasons:

**Assumption 4.** The *only* effects of Smith's intervention will be (a) to alter Smith's psychology in those (and only those) ways necessary to bring it about that he expectably has better post-*T*motives, and (b) consequences of these psychological changes.

**Assumption 5.** Smith can, at *T*, freely choose whether or not to morally enhance himself, and if he chooses to do so, he will make this choice for the best possible reasons (whatever they might be).^21^

Would it be morally permissible for Smith to morally enhance himself in these circumstances? I will argue that, probably, it would.

## Reasons to Enhance

Smith clearly has some moral reason to morally enhance himself: if he does, he will expectably have a better set of motives than he would otherwise have had, and I take it to be uncontroversial that he has some moral reason to bring this result about. (I henceforth omit the ‘moral’ of ‘moral reason’.)

Precisely why he has such reason is open to question. One explanation would run as follows. If Smith brings it about that he expectably has better motives, he expectably brings at least one good consequence about: namely, his having better motives.^22^ And plausibly, we all have at least some moral reason to expectably bring about any good consequence.

This explanation is weakly consequentialist in that it relies on the premise that we have good reasons to expectably bring about any good consequence. But thoroughgoing nonconsequentialists could offer an alternative explanation. They could, for example, maintain that Smith's *act* of moral enhancement has some intrinsic property — such as the property of being an act of self-improvement — that gives him reason to perform it.

But regardless of *why*Smith has reason to morally enhance himself in our scenario, I take it to be intuitively clear that he has such reason. This intuition can, moreover, be buttressed by intuitions about closely related cases. Suppose that some agent Jones is in precisely the same position as Smith, except that in her case, the moral enhancement can be attained not through biomedical means but through some form of self-education — for example, by reflecting on and striving to attenuate her counter-moral emotions. Intuitively, Jones has some reason to morally enhance herself — or so it seems to me. And if pressed on why she has such reason, it seems natural to point to features of her situation that are shared with Smith's — for example, that her morally enhancing herself would have expectably good consequences, or that it may express a concern for the interests of others.^23^

## Reasons Not to Enhance

Smith may also, of course, have reasons not to morally enhance himself, and I now turn to consider what these reasons might be.^24^

### Objectionable Motives

One possibility is that Smith has reason not to enhance himself because he could only do so from some bad motive. I assumed, in setting up the Smith scenario, that if he enhances himself, he will do so from the best possible motive. But the best possible motive may not be good enough.

There are various motives that Smith *could*have for morally enhancing himself. And some of these seem quite unobjectionable: he may believe that he ought to morally enhance himself, he may have a desire to act morally in the future, or he may be moved simply by a concern for the public good. However, we should consider, at this point, an objection due to Michael Sandel. Sandel argues that engaging in enhancement expresses an excessive desire to change oneself, or insufficient acceptance of ‘the given’. And since we have reasons to avoid such motives, we have, he thinks, reasons to refrain from enhancing ourselves.^25^

It would be difficult to deny that Smith's moral enhancement would, like any voluntary instance of enhancement, be driven to some extent by an unwillingness to accept the given (though this need not be his conscious motive). Here, we must agree with Sandel. But what is less clear is that this gives Smith any reason to refrain from enhancement. Leaving aside any general problems with Sandel's suggestion, it faces a specific problem when applied to the case of Smith. Applied to that case, Sandel's claim would be that Smith has reason to accept his bad motives, as well as that which interferes with his good motives. But this is implausible. Surely, if there are any features of himself that he should not accept, his bad motives and impediments to his good motives are among them. The appropriate attitude to take towards such properties is precisely one of *non-acceptance* and a *desire for self-change*.

### Objectionable Means

A second reason that Smith might have not to morally enhance himself is that the biomedical means by which he would do so are objectionable.

We can distinguish between a weak and a strong version of the view that Smith's proposed means are objectionable. On the weak version, his means are objectionable in the sense that it would be *better*if he morally enhanced himself via non-biomedical means. There is certainly some intuitive appeal to this view. It might seem preferable for Smith to enhance himself through some sort of moral training or self-education. When compared with self-education, taking a pill might seem ‘all too easy’ or too disconnected from ordinary human understanding.^26^ Arguably, given the choice between biomedical moral enhancement and moral enhancement via self-education, Smith would have strong reasons to opt for the latter.

Note, however, that Smith's choice is not between alternative means of enhancement, but simply between engaging in biomedical moral enhancement or not. Reasons that Smith has to engage in moral enhancement via other means will be relevant to Smith's choice only to the extent that whether he engages in biomedical moral enhancement will influence the extent to which he seeks moral enhancement through those other means. If Smith's morally enhancing himself through biomedical means would lead him to engage in *less*moral enhancement through some superior means (say, via self-education), then Smith may have some reason not to engage in biomedical moral enhancement. But it is difficult to see why Smith would regard biomedical enhancement and self-education as substitutes in this way. It seems at least as likely that he would regard them as complementary; having morally enhanced himself in one way, he may feel more inclined to morally enhance himself in the other (say, because he enjoys the experience of acting on good motives).

One might, at this point, turn to a stronger version of the ‘objectionable means’ claim, arguing that to adopt biomedical means to moral enhancement is objectionable not just relative to other alternative means, but in an *absolute*sense. Indeed, it is so absolutely objectionable that any moral benefits of Smith's morally enhancing himself would be outweighed or trumped by the moral costs of using biomedical intervention as a means.

Any claim that biomedical means to moral enhancement are absolutely objectionable is likely to be based on a claim that they are unnatural. Certainly, this is a common means-based criticism levelled at biomedical enhancement.^27^ But the problem is to come up with some account of naturalness (or unnaturalness) such that it is true both that:

1. using biomedical means to morally enhance oneself *is*unnatural,

and that:

1. this unnaturalness gives a person reason not to engage in such enhancement.

Can any such account be found?

David Hume distinguished between three different concepts of nature; one which may be opposed to ‘miracles’, one to ‘the rare and unusual’, and one to ‘artifice’.^28^ This taxonomy suggests a similar approach to the concept of unnaturalness. We might equate unnaturalness with miraculousness (or supernaturalness), with rarity or unusualness, or with artificiality. In what follows I will consider whether any of these concepts of naturalness succeeds in rendering both [1] and [2] plausible.

#### Unnaturalness as Supernaturalness

Consider first the concept of unnaturalness as supernaturalness. On one popular account of this concept, something like the following is true: something is unnatural if, or to the extent that, it lies outside the world that can be studied by the sciences.^29^ It seems clear, on this view, that biomedical interventions are not at all unnatural, for such interventions are precisely the sort of thing that *could*be studied by the sciences. The concept of unnaturalness as supernaturalness thus renders [1] clearly false.

#### Unnaturalness as Unusualness

The second concept of unnaturalness suggested by Hume's analysis is that which can be equated with unusualness or unfamiliarity. Leon Kass's idea of unnaturalness as disconnectedness from everyday human understanding may be a variant of this concept.^30^

Unusualness and unfamiliarity are relative concepts in the following way: something has to be unusual or unfamiliar *for* or *to* someone. Thus, whether Smith's biomedical intervention would qualify as unnatural may depend on whom we relativise unusualness and unfamiliarity to. For us inhabitants of the present day, the use of biomedical technology for the purposes of moral enhancement certainly does qualify as unusual and unfamiliar, and thus, perhaps, as unnatural. But for some future persons, it might not. Absent any specification of how to relativise unusualness or unfamiliarity, it is indeterminate whether [1] is true.

We need not pursue these complications, however, since regardless of whether [1] comes out as true on the current concept of unnaturalness, [2] appears to come out false. It is doubtful whether we have any reason to avoid adopting means merely because they are unusual or unfamiliar, or, for that matter, disconnected from everyday human understanding. We may often prefer familiar means to unfamiliar ones on the grounds that predictions about their effects will generally be better informed by evidence, and therefore more certain. Thus, if I am offered the choice between two different drugs for some medical condition, where both are thought to be equally safe and effective, I may choose the more familiar one on the grounds that it will probably have been better studied and thus have more certain effects. But the concern here is not ultimately with the unnaturalness — or any other objectionable feature — of the means, but rather with the effects of adopting it. I will return to the possible adverse effects of Smith's enhancement below. The position I am interested in here is whether the unfamiliarity of some means gives us reasons not to use it *regardless*of its effects. To affirm that it does seems to me to involve taking a stance that is inexplicably averse to novelty.

#### Unnaturalness as Artificiality

Consider finally the concept of unnaturalness as artificiality. This is arguably the most prevalent concept of naturalness to be found in modern philosophy.^31^ It may be roughly characterised as follows: something is unnatural if it involves human action, or certain types of human action (such as intentional action).

Claim [1] is quite plausible on this concept of unnaturalness. Biomedical interventions clearly involve human action — and almost always intentional action. However, [2] now looks rather implausible. *Whenever*we intentionally adopt some means to some end, that means involves intentional human action. But it does not follow that we have reason not to adopt that means. If it did, we would have reason not to intentionally adopt any means to any end. And this surely cannot be right.

The implausibility of [2] on the current concept of unnaturalness can also be brought out by returning to the case where moral enhancement is achieved through self-education rather than biomedical intervention. Such enhancement seems unproblematic, yet it clearly involves unnatural means if unnaturalness is analysed as involving or being the product of (intentional) human action.

We should consider, at this point, a more restrictive account of unnaturalness as artificiality: one which holds that, in order to qualify as unnatural, something must not only involve (intentional) human action, it must also involve *technology*— the products of highly complex and sophisticated social practices such as science and industry. Moving to this account perhaps avoids the need to classify practices such as training and education as unnatural. But it still renders unnatural many practices which, intuitively, we may have no means-based reasons to avoid. Consider, for example, the treatment of disease. This frequently involves biomedical technology, yet it is not clear that we have any means-based reasons not to engage in it. To avoid this problem, the concept of unnaturalness as artificiality would have to be limited still further, such that technology-involving means count as unnatural only if they are not aimed at the treatment of disease. On this view, Smith's means are not unnatural in themselves. Rather the unnaturalness arises from the combination of his means with certain intentions or aims. Perhaps by restricting the concept of unnaturalness in this way, we avoid classifying as unnatural practices (such as self-education, or the medical treatment of diseases) that seem clearly unobjectionable. However, it remains unclear *why*, on this account of the unnatural, we should have reasons to avoid unnatural practices. In attempting to show that Smith has reason not to engage in biomedical moral enhancement, it is not enough to simply stipulate some concept of unnaturalness according to which his engaging in moral enhancement comes out as unnatural while seemingly less problematic practices come out as natural. It must be shown that a practice's being unnatural *makes* it problematic, or at least provides evidence for its being problematic. Without such a demonstration, the allegation of unnaturalness does no philosophical work, but merely serves as a way of asserting that we have reasons to refrain from biomedical moral enhancement.

#### Objectionable Means?

I have argued that none of the three concepts of unnaturalness suggested by Hume's analysis renders both [1] and [2] plausible. If my conclusions are correct, it follows that none of these concepts of unnaturalness point to any means-based reason for Smith to refrain from moral enhancement. There may be some further concept of unnaturalness on the basis of which one could argue more convincingly for [1] and [2]. Or there may be some way of showing that biomedical moral enhancement involves means that are objectionable for reasons other than their unnaturalness. But I am not sure what the content of these concepts and arguments would be.

### Objectionable Consequences

Would the consequences of Smith's enhancement provide him with reasons to refrain from engaging in that enhancement? Two points about this possibility need to be noted up front. First, since we are assuming that Smith's moral enhancement will have no side-effects (Assumption 4), the only consequences that his action will have are:

- That he will expectably have better post-*T* motives than he would otherwise have had
- Those, and only those, psychological changes necessary to bring about (a)
- Consequences that follow from (a) and (b)

Thus, if Smith has consequence-based reasons to avoid moral enhancement, those reasons must be grounded on the features — presumably the intrinsic badness — of (a), (b) or (c).

Second, there are some moral theories which constrain whether, or to what extent, consequences (a) and (c) could be bad. Consider theories according to which only hedonic states (such as states of pleasure or pain) can be intrinsically good or bad. On these theories, (a) could not be intrinsically bad since motives are not hedonic states. Consider, alternatively, a consequentialist moral theory according to which the moral goodness of a motive is determined by the goodness of the consequences of a person's having it. On this theory, if Smith indeed has better post-*T* motives, then the consequences of his having those motives — these fall under (c) — must be better than the corresponding consequences that would have come about had he had worse motives. Smith's having better motives is guaranteed to have better consequences than his having worse motives because having good consequences is what makes a motive good. In what follows, I will assume, for the sake of argument, that moral theories which limit the possible badness of (a) and (c) in these ways are false.

#### Identity Change

One bad effect of Smith's morally enhancing himself might be that he loses his identity. Worries about identity loss have been raised as general objections to enhancement, and there is no obvious reason why they should not apply to cases of moral enhancement.^32^

Clearly, moral enhancement of the sort we are considering need not be identity-altering in the strong sense that Smith will, post-enhancement, be a different person than he was before. Our moral psychologies change all the time, and sometimes they change dramatically, for example, following particularly traumatic experiences. When these changes occur, we do not think that one person has literally been replaced by another. However, perhaps Smith's moral enhancement would be identity-altering in the weaker sense that it would change some of his most fundamental psychological characteristics — characteristics that are, for example, central to how he views himself and his relationships with others, or that pervade his personality.^33^

Suppose we concede that Smith's moral enhancement would be identity altering in this weaker sense. This may not give Smith any reason to refrain from undergoing the change. Plausibly, we have reasons to preserve our fundamental psychological characteristics only where those characteristics have some positive value. But though Smith's counter-moral emotions *may*have some value (Smith may, for example, find their experience pleasurable), they need not.

#### Restricted Freedom

By morally enhancing himself Smith will bring it about that he has better post-*T* motives, taken in sum, than he would otherwise have had. However, it might be thought that this result will come at a cost to his freedom: namely, he will, after *T*, lack the freedom to have and to act upon certain bad motives. And even though having and acting upon bad motives may itself have little value, it might be thought that the *freedom* to hold and act upon them *is*valuable. Indeed, this freedom might seem to be a central element of human rational agency. Arguably, Smith has reasons not to place restrictions on this freedom.

The objection that I am considering here can be captured in the following two claims:

1. Smith's morally enhancing himself will result in his having less freedom to have and to act upon bad motives.
2. Smith has reason not to restrict his freedom to have and act upon bad motives.

Claim [4] is, I think, problematic. It is not obvious that the freedom referred to therein has any value. Moreover, even if this freedom does have value, there may be no problem with restricting it provided that the restriction is itself self-chosen, as in Smith's case it is. However, I will focus here on [3]. The proponent of [3] is committed to a certain understanding of freedom. She would have to maintain that freedom consists not merely in the absence of external constraints, but also in the absence of internal psychological constraints, for it is only Smith's internal characteristics that would be altered by his moral enhancement. This view could be sustained by regarding the self as being divided into two parts — the true or authentic self, and a brute self that is external to this true self. One could then regard any aspect of the brute self which constrains the true self as a constraint on freedom.^34^ And one could defend [3] on the ground that Smith's enhancement will alter his brute self in such a way that it will constrain his autonomous self.

There would be some justification for thinking that Smith's moral enhancement would alter his brute self rather than his true self. We are assuming that Smith's enhancement will attenuate certain emotions, so it will presumably work by altering the brain's emotion-generating mechanisms, and these mechanisms are arguably best thought of as part of the brute self. Certainly, it would be strange to think of the predominantly subconscious mechanisms which typically call forth racial aversion or impulsive aggression as part of the true autonomous self.

However, the view that moral enhancement would alter Smith's brute self in a way that would *interfere with*his autonomous self seems to be at odds with my assumption (Assumption 3) about the mechanism of that enhancement. Since Smith's enhancement is assumed to attenuate certain emotions, it presumably works by *suppressing* those brute mechanisms that generate the relevant emotions. The enhancement seems to work by *reducing*the influence of Smith's brute self and thus allowing his true self *greater*freedom. It would be more accurate to say that the enhancement increases Smith's freedom to have and to act upon good motives than to say that it diminishes his freedom to have and to act upon bad ones.

#### Inducing Free-riding

The final possibility that I want to consider is that Smith might have reason to refrain from moral enhancement because his having better motives would induce others to free-ride.

Why this might occur can be illustrated through the following scenario. Suppose that Jack and Jim are the only fishermen who work in a certain bay. Fish stocks have become depleted in the bay, and both would prefer it if the stocks rose, but neither wants to limit his or her catch. Nevertheless, they formulate a plan: they will for the next month limit themselves to a quota of twenty fish each per day — significantly fewer than either would otherwise expect to take even with depleted stocks.

**Figure 1:**
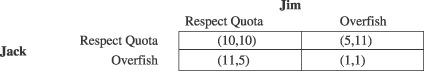
Jack & Jim.

Each fisherman can either stick to the plan (‘respect the quota’) or not (‘overfish’). There are, then, four possible action-pairs (Jack respects the quota, Jim overfishes; Jim respects the quota, Jack overfishes; *et cetera*). The payoffs — measured in terms of goodness — for each fisherman from each of these action-pairs are depicted in Figure 1 below. They have been chosen to reflect the fact that each fisherman's payoff is negatively correlated with the extent to which future stocks are depleted, but positively correlated with the number of fish caught by himself in the present.^35^

**Figure 2:**
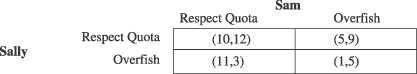
Sally & Sam.

Suppose that neither fisherman can observe the number of fish caught by the other, but each can observe the other's motives. Suppose further that Jim's motives are *Self-Interested*, meaning that he always does whatever maximises his own payoff, whereas Jack's are either *Morally Good*— meaning that he always sticks to the plan, respecting the quota — or *Morally Bad*— meaning that he always reneges on the plan, overfishing.

If Jack's motives are *Morally Bad*, then Jim will know that Jack will overfish. He will thus face a choice between respecting the quota and getting a payoff of 5, or overfishing and getting a payoff of 1. Since his motives are *Self-Interested*, Jim will respect the quota.

But now suppose that Jack's motives are *Morally Good.*Jim will thus know that Jack will overfish. Hence, he faces a choice between respecting the quota, and getting a payoff of 10, or overfishing, and getting a payoff of 11. Having *Self-Interested*motives, Jim will overfish. By having *Morally Good*motives, rather than *Morally Bad*ones, Jack induces Jim to overfish rather than respecting the quota. That is, he induces Jim to take advantage of his good motives in a way that harms him, reduces their combined payoff, and disrespects their earlier agreement.

This is a rather stylised scenario. Nevertheless, it illustrates one way in which one person's having better motives could, by altering the payoff structure faced by others, induce those others to free-ride in ways that might well be regarded as morally bad.

However, just as we can construct scenarios in which one person's having good motives induces another to act badly, so too we can construct scenarios in which it has the opposite effect. Consider a variant of the Jack & Jim scenario in which Sally and Sam face a similar problem but this time with the following payoffs:

Sally's payoffs are the same as Jack's and Jim's, but Sam's payoffs have changed to reflect that he has a slightly different value function over future fish population.^36^ Assume again that the fishermen cannot observe each other's catch, but can observe one another's motives. Assume also that Sam has *Self-Interested*motives. Then if Sally has *Morally Bad*motives, so that she always overfishes, Sam will face a choice between respecting the quota and getting a payoff of 1 or overfishing and getting a payoff of 5. Having *Self-Interested*motives, Sam will overfish. On the other hand, if Sally has *Morally Good*motives, so that she always respects the quota, Sam will have a choice between respecting the quota and getting a payoff of 10, or overfishing and getting a payoff of 9. He will respect the quota.

We thus have an interaction in which one person's having better motives induces the other *not*to free-ride. The effect is the opposite of that seen in the Jack & Jim scenario. There are also, of course, many collective action problems in which a change in one person's motives will simply have no effect on whether the other free-rides. (The standard Prisoners’ Dilemma is an example. A self-interested agent will always free-ride in this scenario.)

Smith's moral enhancement may reduce his own inclination to free-ride in many situations. But the foregoing discussion suggests that it will increase the inclination of others to free-ride only in a subset of these cases. It thus seems unlikely that his enhancement would lead to a net increase in free-riding.

## Implications

I have argued that Smith has some reason to morally enhance himself via biomedical means. I have also rejected several arguments for the existence of good countervailing reasons. Thus, I hope that I have offered some support for the claim that it would be morally permissible for Smith to engage in biomedical moral enhancement. But if it would be permissible for Smith to morally enhance himself, then the Bioconservative Thesis is almost certainly false. For as I claimed earlier, it is plausible that biomedical moral enhancement technologies will become technically feasible in the medium term future. And it is almost certain that, if they do become feasible, some — probably many — actual future people will find themselves in scenarios sufficiently like Smith's that our conclusions about Smith will apply to them also: contrary to the Bioconservative Thesis, there will be people for whom it would be morally permissible to engage in biomedical enhancement.

I should end, however, by noting that the Bioconservative Thesis is not the only claim advanced by the opponents of enhancement. As well as claiming that it would not be morally permissible for people to enhance themselves, many bioconservatives would assert that it would not be permissible for us to *develop*technologies for enhancement purposes, nor for us to *permit*enhancement. For all that I have said, these claims may well be true. It would not follow straightforwardly from the fact that it would be permissible for some future people to morally enhance themselves — given the presence of the necessary technology and the absence of legal barriers — that they could permissibly be allowed to do so, or that we could permissibly develop the technologies whose availability we are taking as given. Other factors would need to be considered here. It may be, for example, that if we were to develop moral enhancement technologies, we would be unable to prevent their being used in undesirable ways — for example, to enhance self-interestedness or *im*morality. Whether we could permissibly develop or permit the use of moral enhancement technologies might thus depend on a weighing of the possible good uses of those technologies against the possible bad ones.

## NOTES

1 M. Verroken, ‘Drug use and abuse in sport’, in D. R. Mottram (ed.) *Drugs in Sport* (London: Routledge, 2005), pp. 29–63.

2 B. Tindall, ‘Better playing through chemistry’, *New York Times*, 17 Oct 2004.

3 L. D. Johnston, P. M. O'Malley & J. G. Bachman, *Monitoring the Future National Survey Results on Drug Use, 1975–2002: II. College Students and Adults Ages 19–40* (Washington DC: US Department of Health and Human Services, 2003); TeterC JMcCabeS ELaGrangeK‘Illicit use of specific stimulants among college students: prevalence, motives, and routes of administration’Pharmacotherapy200626101501151010.1592/phco.26.10.1501PMC179422316999660.

4 LynchG‘Memory enhancement: the search for mechanism-based drugs’Nature Neuroscience200251035103810.1038/nn93512403980; ScottRBourtchouladzeRGossweilerS‘CREB and the discovery of cognitive enhancers’Journal of Molecular Neuroscience2002191711771221277710.1007/s12031-002-0029-z; TullyTBourtchouladzeRScottR‘Targeting the CREB pathway for memory enhancers’Nature Reviews Drug Discovery2003226727710.1038/nrd106112669026.

5 YesavageJMumenthalerMTaylorJ‘Donezepil and flight simulator performance: effects on retention of complex skills’Neurology2001591231251210532010.1212/wnl.59.1.123.

6 CaldwellJ ACaldwellJ LSmytheN K‘A double-blind, placebo-controlled investigation of the efficacy of modafinil for sustaining the alertness and performance of aviators: a helicopter simulator study’Psychopharmacology20001502722821092375510.1007/s002130000450; TurnerD CRobbinsT WClarkLAronA RDowsonJSahakianB J‘Cognitive enhancing effects of modafinil in healthy volunteers’Psychopharmacology20031652602691241796610.1007/s00213-002-1250-8.

7 Some writers may be opposed only to certain kinds of enhancement, but others appear to find *all*enhancement problematic, and perhaps impermissible, preferring that biomedical technology is used only maintain and restore health. The most prominent recent exponent of this view is Michael Sandel. See SandelM J‘The case against perfection’The Atlantic Monthly20042933506515468473; and his *The Case Against Perfection: Ethics in the Age of Genetic Engineering* (Cambridge, MA: Harvard University Press, 2007) esp. at pp. 12, 47–49.

8 See, for example, G. A. Annas, ‘Cell division’, *Boston Globe*, 21 April 2002; F. Fukuyama, *Our Posthuman Future: Consequences of the Biotechnology Revolution* (New York: Farrar, Straus, and Giroux, 2002), p. 97; B. McKibben, ‘Designer genes’, *Orion*, 30 April 2003; MehlmanM J‘Genetic enhancement: plan now to act later’Kennedy Institute of Ethics Journal200515177821588179710.1353/ken.2005.0001.

9 For competitiveness-based objections to enhancement, see A. Buchanan, D. Brock, N. Daniels & D. Wikler, *From Chance to Choice: Genetics and Justice* (Cambridge: Cambridge University Press, 2000), pp. 188–191; McKibben (2003) op. cit.; FarahM JIllesJCook-DeeganR‘Neurocognitive enhancement: what can we do and what should we do?’Nature Reviews Neuroscience2004542142510.1038/nrn139015100724 at p. 423; Sandel (2007) op. cit., pp. 8–12. For discrimination based objections, see, for example, Sandel (2007) op. cit., p. 15.

10 I focus on the morality of motives because I take this to be common ground. Some Kantians might deny that acts or behaviour are the proper objects of moral appraisal, and some of those who regard acts as the most basic units of moral appraisal might shy away from making judgments of moral character. But I think that all, or nearly all, would accept that motives come in varying degrees of morality, even if their morality derives ultimately from the behaviour that they motivate or the virtues they derive from or constitute.

11 Immanuel Kant is of course the classic exponent of this of view, claiming as he did that the only thing ‘good in itself’ is a good will, where the good will is understood as the capacity to engage in operations of practical reason that are governed in the right way by the moral law. See especially his *Critique of Practical Reason*, 3rd edn., L. W. Beck, trans. (New York: Macmillan, 1993) Book I, Chapter I.

12 See, for example, J. S. Mill, *Utilitarianism* G. Sher, ed. (Indianapolis: Hackett, 1979), pp. 27–28.

13 The classic exposition of this view comes from Aristotle. See his *Nicomachean Ethics*, T. Irwin, trans. (Indianapolis: Hackett, 1985).

14 I assume, for argument's sake, that reasoning does not itself involve the emotions. For a denial of this view, see Antonio Damasio, *Descartes’ Error: Emotion, Reason and the Human Brain* (London, Vintage, 2006).

15 Most obviously, this would be held by mind-body parallelists who believe that mind and brain are causally insulated from one another. The most famous exponent of this view is G. W. Leibniz. See ‘New system, and explanation of the new system’ in his *Philosophical Writings*, G. H. R. Parkinson, ed., M. Morris, trans. (London: Dent, 1973).

16 CroweR R‘An adoption study of antisocial personality’Archives of General Psychiatry197431785791444124610.1001/archpsyc.1974.01760180027003; CadoretR J‘Psychopathology in adopted-away offspring of biologic parents with antisocial behavior’Archives of General Psychiatry19783517618462350410.1001/archpsyc.1978.01770260054005; GroveW MEckertE DHestonL‘Heritability of substance abuse and antisocial behavior: a study of monozygotic twins reared apart’Biological Psychiatry19902712931304236411810.1016/0006-3223(90)90500-2.

17 BrunnerH GNelenM RBreakefieldX O‘Abnormal behaviour associated with a point mutation in the structural gene for Monoamine Oxidase A’Science19932625133578580821118610.1126/science.8211186; BrunnerH GNelenM Rvan ZandvoortP‘X-linked borderline mental retardation with prominent behavioural disturbance: phenotype, genetic localization, and evidence for disturbed monoamine metabolism’American Journal of Human Genetics1993526103210398503438PMC1682278.

18 CaspiAMcClayJ‘Evidence that the cycle of violence in maltreated children depends on genotype’Science20022978518541216165810.1126/science.1072290; de AlmeidaR M MFerariP FParmigianiS‘Escalated aggressive behavior: Dopamine, serotonin and GABA’European Journal of Pharmacology200552651641632564910.1016/j.ejphar.2005.10.004.

19 HartA JWhalenP JShinL M‘Differential response in the human amygdala to racial outgroup vs. ingroup face stimuli’Neuroreport: For Rapid Communication of Neuroscience Research2000112351235510.1097/00001756-200008030-0000410943684; PhelpsE AO’ConnorK JCunninghamW A‘Performance on indirect measures of race evaluation predicts amygdala activation’Journal of Cognitive Neuroscience2000127297381105491610.1162/089892900562552; CunninghamW AJohnsonM KRayeC L‘Separable neural components in the processing of black and white faces’Psychological Science2004158068131556332510.1111/j.0956-7976.2004.00760.x.

20 I also assume, as a background to the listed assumptions, that Smith is a normal person living in a world similar to our own — that is, a world governed by the scientific and social scientific principles that we take to govern our own world.

21 I take it that Assumption 5 entails at least that there is no physical or legal constraint on Smith's morally enhancing himself.

22 Smith might also bring many other expectably good consequences about — for example, those that follow from his expectably having good motives.

23 I do not claim that Smith's reason to engage in moral enhancement is as strong as Jones's.

24 The reasons considered in this section are based on a range of different substantive moral views. I do not claim that there is any one moral viewpoint which could accommodate all of the putative reasons discussed.

25 Sandel (2004) op. cit., pp. 50–65; Sandel (2007) op. cit.

26 See KassL R‘Ageless bodies, happy souls: biotechnology and the pursuit of perfection’The New Atlantis2003192815584192 at pp. 21–24; and President's Council on Bioethics, *Beyond Therapy: Biotechnology and the Pursuit of Happiness* (Washington DC: President's Council on Bioethics, 2003) at pp. 290–293.

27 See, for example, Kass (2003) op. cit., pp. 17, 20–24; President's Council on Bioethics (2003) op. cit., pp. 290–293.

28 D. Hume, *A Treatise of Human Nature*, L. A. Selby-Bigge, ed. (Oxford: Clarendon, 1888), pp. 473–475.

29 See, for example, G. E. Moore, *Principia Ethica*(Cambridge: Cambridge University Press, 1903), p. 92.

30 Kass (2003) op. cit., pp. 22–24.

31 HeydD‘Human nature: an oxymoron?’Journal of Medicine and Philosophy20032821511691294321210.1076/jmep.28.2.151.14205 at pp. 156–157.

32 See, for example, WolpeP R‘Treatment, enhancement, and the ethics of neurotherapeutics’Brain and Cognition2002503873951248048510.1016/s0278-2626(02)00534-1 at pp. 393–394; President's Council on Bioethics (2003) op. cit., p. 294.

33 See, for a discussion of this weaker sense of ‘identity’, M. Schechtman, *The Constitution of Selves* (Ithaca, NY: Cornell University Press, 1996), esp. at pp. 74–76.

34 See, for an example of this approach, C. Taylor, ‘What's wrong with negative liberty’ in A. Ryan (ed.) *The Idea of Freedom* (London, Oxford University Press, 1979), pp. 175–193.

35 These payoffs are generated from the following assumptions. Assume that if each fisherman respects the quota, the fish population will be high by the end of the month, if only one respects the quota, the population will be low, and if neither respects the quota, the population will be very low. The value to each of having high population is 16, of having a low population is 11, and of having a very low population is 1. Suppose, moreover, that the short-term value to each of respecting the quota is −6 and the value of not doing so is 0. Thus, the value to one neighbour of respecting the quota when the other also respects the quota is 16 − 6 = 10, of respecting the quota when the other overfishes is 11 − 6 = 5, of overfishing when the other respects the quota is 11 − 0 = 11, and of overfishing when the other also overfishes is 1 − 0 = 1.

36 For Jack, Jim and Sally, the value of a low future fish population is much greater than that of a very low population, but there is little further gain from having a high rather than a low population (see *supra*n. 35). For Sam, having a high population has a much higher value than having a low population, whereas there is relatively little difference in value between a low and a very low population. The payoffs for Sam presented in the table reflect the following underlying values: value of a high fish population, 18; value of a low fish population, 9; value of a very low fish population, 5; short-term value of respecting the quota, −6; short-term value of overfishing, 0.

